# Social inhibition in depressed patients is associated with an altered activation profile of the interleukin-6-inducible transcription factor STAT3

**DOI:** 10.1016/j.bbih.2025.100968

**Published:** 2025-02-18

**Authors:** Katharina von Knebel, Julia Staab, Anke Gregus, Linus Remling, Oliver Wirths, Carsten Spitzer, Christoph Herrmann-Lingen, Holger M. Reichardt, Thomas Meyer

**Affiliations:** a Department of Psychosomatic Medicine and Psychotherapy, Georg-August University, Göttingen, Germany; b Department of Psychiatry and Psychotherapy, Georg-August University, Göttingen, Germany; c Institute for Cellular and Molecular Immunology, Georg-August University, Göttingen, Germany; d Department of Psychosomatic Medicine and Psychotherapy, University Medical Center Rostock, Rostock, Germany

**Keywords:** Depressed patients, Effects of psychotherapy, Social inhibition, STAT3 activation, Type D personality

## Abstract

**Objective:**

Numerous studies have described the role of STAT3 (signal transducer and activator of transcription 3) in infections, but little is known on whether this transcription factor is linked to negative affectivity (NA) and social inhibition (SI), leading to social withdrawal as a typical symptom of various infections.

**Methods:**

In this study, we isolated peripheral blood mononuclear cells (PBMCs) from 63 consecutive depressed patients (mean age 41.4 ± 16.1 years; 40 females) before and after psychotherapeutic intervention and measured STAT3 tyrosine phosphorylation (pSTAT3) with and without *in vitro* interleukin-6 (IL-6) stimulation of these cells using flow cytometry. In addition, all study participants were assessed for NA and SI using the German version of the Type D Scale-14 (DS-14) questionnaire with a cut-off level of ≥10 for each subscale.

**Results:**

While NA was unrelated to STAT3 activity, PBMCs from SI-positive patients had an increased baseline STAT3 activation level, which made the cells less sensitive to *in vitro* IL-6 stimulation (11.5% vs. 9.1%, p = 0.036). The stimulatory capacity, defined as the difference in pSTAT3 levels from IL-6-stimulated to unstimulated cells during hospitalization, was significantly lower in PBMCs from SI-positive than from SI-negative patients (−1.7% vs. 6.6%, p = 0.007). The sensitivity of PBMCs to IL-6 stimulation was negatively correlated with the SI score (r = −0.295, p = 0.019). Of note, the altered sensitivity to STAT3 phosphorylation remained stable, when adjusted for clinically relevant confounders in multivariate analysis (Exp(β) = 0.891, 95%-confidence interval = 0.804–0.988, p = 0.029).

**Conclusion:**

These findings point towards a possible relationship between STAT3 signaling and social inhibition in depressed patients.

## 1 Introduction

Major depressive disorder (MDD), characterized by pervasive low mood, anhedonia and reduced self-esteem, is among the most common mental health conditions. The World Health Organization estimates that over 322 million people worldwide suffer from depression, with women being affected 1.5 to 2 times more often than men (Bromet et al., 2011; Eid et al., 2019), and it is projected to be the leading cause of disability by 2030 (World Health Organization, 2012; Chan et al., 2024). In the early 1990s, a putative link between depressive disorders and the activation of the peripheral immune system was first described (Smith, 1991; Maes, 1995). Today, further evidence suggests that inflammation plays a critical role in the pathogenesis of depression (Miller and Raison, 2016; Roohi et al., 2021). Numerous studies have found that MDD patients have increased serum concentrations of pro-inflammatory cytokines, such as interleukin-1β (IL-1β), IL-6, and tumor necrosis factor-α (TNFα) (Dowlati et al., 2010; Köhler et al., 2017; Kwon et al., 2017; Çakici et al., 2020; Reisinger et al., 2021). However, there is little knowledge regarding the pathophysiological mechanisms that control inflammatory processes in the context of depressive symptoms.

An essential component of depression and depressive-like behaviours is negative affectivity (NA), which is defined as the tendency to experience negative emotions. In combination with social inhibition (SI), which is a trait-like tendency to inhibit self-expression in social interactions, NA constitutes the type D (*“*distressed*”*) personality (Denollet, 1998, 2005; Pedersen and Denollet, 2003; Michal et al., 2011; Park et al., 2014). Type D patients suffering from cardiovascular diseases have elevated levels of pro-inflammatory cytokines (e.g. IL-6 and TNFα including its receptor) compared to non-type D individuals (Conraads et al., 2006; Wang et al., 2022; Yi et al., 2022). However, van Dooren and colleagues found no association between inflammatory parameters such as IL-6, TNFα, or high-sensitivity CRP and type D (van Dooren et al., 2016).

It is well known that the intracellular effects of IL-6 and other cytokines are mediated by the transcription factor STAT3 (signal transducer and activator of transcription 3), which becomes phosphorylated by Janus kinases (JAKs) at a singular, carboxy-terminal tyrosine residue (Y705) following recruitment to the activated IL-6 receptor (Zhong et al., 1994). Upon binding of the pro-inflammatory IL-6 to its cell surface receptor, tyrosine-phosphorylated STAT3 (pSTAT3) is transmitted to the nucleus, where it is sequence-specifically recruited to promoter regions in cytokine-responsive target genes to modulate their transcription. The transcription factor STAT3 was selected as a target in this analysis because serum levels of IL-6 are known to be elevated in depressed patients and, furthermore, tyrosine phosphorylation of STAT3 is induced by the binding of the cytokine IL-6 to its cell surface receptor (Zhong et al., 1994; Kwon et al., 2017; Reisinger et al., 2021).

Using proteasomal data, Gong et al. recently demonstrated that social isolation and loneliness, which are both characterized by a lower frequency of in-person social contacts, have similar protein signatures in common, including necrosis, cell death regulation, caspase activation, malignant transformation, and host vulnerability (Gong et al., 2025). Interestingly, all these biological pathways are under transcriptional regulation by STAT proteins (Doudin et al., 2023), suggesting that these inflammatory proteins may be the missing molecular link between cytokine activation and social deficit behaviours such as loneliness and social isolation. Viral, bacterial and parasitic infections lead to activation of STAT proteins and, furthermore, infectious diseases decrease the size of social networks by inducing social withdrawal. In the case of an ongoing infection, the act of being physically isolated from peers or family members may not only reduce the transmission of the pathogen, but also increases the chance of survival of close relatives and peers.

The present study in depressed patients focused on the association between psychometrically assessed SI and pSTAT3 levels, which was used as an intracellular marker of immune cell activation. We tested the hypothesis that pSTAT3 activation is related to social deficit behaviours such as SI and loneliness. To this end, pSTAT3 levels were measured in peripheral blood mononuclear cells (PBMCs) isolated from depressed patients on admission and discharge from hospital. The study investigated the potential associations of the psychometrically assessed type D components NA and SI with pSTAT3 levels used as an intracellular marker of immune cell activation in individuals with depressive syndrome.

## 2 Materials and methods

### 2.1 Recruitment of patients

The prospective Cytokine-Induced Transcription in Depressed Inpatients undergoing Psychotherapy (CitDip) study was conducted from February to August 2017 at the Department of Psychosomatic Medicine and Psychotherapy at the University Medical Centre Göttingen, Germany, together with the Asklepios Clinic Tiefenbrunn in Rosdorf, Germany, and registered under the ClinicalTrials.gov ID NCT06388486. The study protocol was carried out in accordance with the Helsinki Declaration and was approved by the local ethics committee of the University of Göttingen. Study participants were 18 years or older and provided their written informed consent. All consecutive patients included in the study were diagnosed with current depression according to DSM-IV criteria and were admitted for inpatient psychotherapy. Patients were excluded if they had inadequate knowledge of the German language, were unable to give informed consent due to the presence of predefined diagnosed psychiatric comorbidities such as drug abuse or psychotic diseases or had incomplete psychometric assessment at baseline. Furthermore, patients were ineligible if they were deemed clinically unstable by their treating physician. Two patients had to be excluded due to either missing data in the psychometric assessment or a later diagnosis of borderline syndrome during hospitalization, which was an exclusion criterion, leaving a total of n = 63 study participants for analysis. During their hospital stay, all patients received psychotherapy in a multiprofessional clinical setting, including individual sessions and group therapy. At baseline, data from patients’ records including demographics, medical history and somatic comorbidities were assessed. Previous cardiovascular history (e.g. myocardial infarction, coronary heart disease, stroke) and chronic diseases were obtained from the medical records. In addition to the current medication, cardiovascular risk factors, including hypertension, diabetes mellitus, obesity, and smoking status, were documented. A priori sample size estimation was performed using G∗Power software with an effect size of ρ = 0.4, an α-error probability of α = 0.05, and a power (1-β) of p = 0.95, resulting in a total calculated sample size of 59 study participants.

### 2.2 Psychometric assessment

At baseline before undergoing psychotherapeutic intervention and five to six weeks later at follow-up, patients were asked to complete the German version of the Hospital Anxiety and Depression Scale (HADS) and the Beck Depression Inventory (BDI) as part of the routine diagnostic procedure (Beck and Beck, 1972; Zigmond and Snaith, 1983; Herrmann, 1997). The HADS is a self-administered questionnaire including 14 items scored on a four-point Likert scale split into two sub-scales of depression (HADS-D) and anxiety (HADS-A), each consisting of seven multiple-choice items covering cognitive-affective features of depression and generalized anxiety (Bjelland et al., 2002). Furthermore, type D personality was assessed by using the German version of the Type D Scale-14 (DS-14) questionnaire (Denollet, 2005; Grande et al., 2010). Scores were calculated separately for the two dimensions of type D personality, i.e. NA and SI, with seven five-point Likert-scaled items ranging from 0 = false to 4 = true for each subscale. The NA items cover feelings of dysphoria, worries, and irritability, whereas the seven SI items ask about discomfort in social interactions, reticence, and social poise. Scores ≥10 in both subscales (NA and SI) constitute type D personality. The HADS and DS-14 instruments used in this study have been shown to be reliable and valid (Denollet, 2005; Michal et al., 2011). In our analysis, we also tested for associations with feelings of loneliness using the slightly modified, German version of the UCLA Loneliness Scale, which is a short, 18-item general measure of loneliness (Windisch and Kniel, 1988; Russell, 1996). The Flourishing Scale is a brief 8-item summary measure of the participant's self-perceived success in important areas such as relationships, self-esteem, autonomy, resilience, and optimism with a Cronbach α coefficient of 0.87 suggesting adequate internal item consistency (Diener et al., 2010; Esch et al., 2013).

### 2.3 Blood samples

Blood samples were collected at baseline and follow-up by venipuncture of an arm vein. Heparinized and EDTA plasma was obtained by immediate centrifugation and stored at −80 °C until being analysed in the central laboratory of the University Medical Centre Göttingen. Additionally, PBMCs were isolated for subsequent FACS analysis by density centrifugation of PBS-diluted blood samples (1:2) using a Ficoll-Hypaque gradient (Histopaque-1077, Sigma-Aldrich, Taufkirchen, Germany), according to the manufacturer's instructions. Approximately 5 x 10^6^ PBMCs were recovered from each blood sample and stored at −80 °C in Roswell Park Memorial Institute (RPMI) medium, complemented with 50% fetal bovine serum (FBS) and 10% dimethyl sulfoxide (DMSO). For immunoblotting, proteins from whole cell extracts were dissolved in Laemmli sample buffer and separated by electrophoresis with 10% polyacrylamide gels, followed by electrophoretic transfer to polyvinylidene fluoride (PVDF) membranes. Immunostaining of the denatured proteins was performed after incubation of the membranes with either anti-phospho-STAT3 (D3A7, Cell Signaling, Leiden, The Netherlands), anti-NF-κB p65 (D14E12, Cell Signaling) or anti-glyceraldehyde-3-phosphate dehydrogenase (αGAPDH; 14C10, Cell Signaling) primary rabbit monoclonal antibodies, in combination with an IRDye 800CW-conjugated, polyclonal goat-anti-rabbit secondary antibody (LI-COR).

### 2.4 Flow cytometry

PBMCs were seeded in 12 well plates at 1.0–1.5 x 10^6^ cells/well in 1 ml of RPMI medium supplemented with 10% FBS. Cells were stimulated by adding 50 ng/ml of IL-6 (Biomol) for 30 min or left unstimulated. Subsequently, cells were washed with FACS buffer (BD Biosciences, Heidelberg, Germany) before fixation with BD lyse/fix buffer (BD Biosciences) for 10 min at 37 °C. Cells were permeabilized by slowly adding BD FACS Perm buffer III (BD Biosciences) while vortexing. After incubation for 30 min at −20 °C, cells were washed twice and then resuspended in BD FACS stain buffer (BD Biosciences). Staining was performed for 20 min at room temperature with a rabbit phycoerythrin-conjugated anti-STAT3 monoclonal antibody (BD Biosciences). Flow cytometric analysis was performed using a BD FACS Canto II (BD Biosciences) device. Data analysis was achieved using Flow-Jo (Treestar) version 7.6.5.

### 2.5 Statistical analysis

The cohort of patients was dichotomized into an SI-negative and SI-positive group according to their psychometric assessment from the DS-14 questionnaire. Demographic and clinical data are presented as means and standard deviations for continuous variables or frequencies and percentages for categorical variables. Group comparisons between probands dichotomized along their SI positivity were performed using χ^2^ tests for categorical variables and Student's *t*-test for continuous measures. Pearson's correlation coefficients were calculated to establish possible associations between STAT3 phosphorylation and the SI score in the total study cohort. To test whether the phosphorylation status of STAT3 was independently related to the presence of social inhibition, multiple logistic regression models were calculated by entering sex, age, diabetes mellitus, and hypertension as potentially confounding variables. Except for sex and age, only those variables were included in the models, which in univariate analysis differed significantly between the SI-positive and -negative group. Values are given as Exp(β), corresponding to the odds ratio for the assignment to the SI-positive group, and its confidence interval (95%-CI). This statistical approach was chosen, because STAT3 phosphorylation was determined only at baseline and follow-up, and repeated measurements at multiple time points during psychotherapy were not performed. All statistical analyses were carried out using SPSS 26 (IBM, Armonk, NY, USA). A p value < 0.05 was regarded as statistically significant.

## 3 Results

### 3.1 Baseline characteristics of the total study cohort

Study participants were predominantly female (63.5%), and the mean age was 41.4 ± 16.2 years. As shown in Table 1, our study cohort included 49 individuals (77.8%), who displayed the social inhibition trait. Most study participants were classified as positive for type D personality (73%). Study participants positive for SI differed significantly from SI-negative patients with respect to fewer cases of diabetes mellitus (2.0% in the SI-positive group compared to 28.6% in the SI-negative group, p = 0.001) and their lower prevalence of hypertension (24.5% vs. 57.1%, p = 0.021). Other comorbid diseases or somatic factors did not differ significantly between the two groups. The severity of depressive symptoms decreased significantly across the study cohort from baseline to follow-up, as measured by changes in the HADS (11.8 ± 4.0 vs. 8.8 ± 5.0, p < 0.001) and BDI (28.6 ± 11.2 vs. 20.8 ± 12.7, p < 0.001). These reductions in HADS and BDI scores for depression during hospitalization correlated strongly with each other (r = 0.612, p < 0.001) and indicated a positive effect of psychotherapy. Remarkably, the time-dependent decrease in BDI scores was higher in SI-positive patients than in SI-negative patients (9.3 ± 1.4 vs. 2.3 ± 8.6, p = 0.008), as was also observed, albeit not significantly, for HADS depression scores (3.5 ± 4.3 vs. 1.8 ± 4.2, p = 0.111). The use of antidepressants did not differ between the two groups, with 5 SI-negative patients (36%) and 18 SI-positive patients (37%) taking these medications (p = 0.944). However, the decrease in depression severity was not associated with STAT3 stimulability parameters (p ≥ 0.153), nor did it differ between the two groups of SI-positive and -negative SI patients (p = 0.456).

**Table 1:** Tyrosine-phosphorylated STAT3 (pSTAT3) in peripheral blood mononuclear cells (PBMCs) in the entire study cohort as well as the two subgroups with and without social inhibition (SI), classified according to the SI dimension of the DS-14 questionnaire. Isolated cells were either unstimulated or stimulated *in vitro* with IL-6 at baseline (BL) and follow-up (FU). Data show mean values including standard deviations of flow cytometric measurements in brackets. Significant changes are highlighted in bold.

	Total Cohort (n = 63)	SI neg. (n = 14)	SI pos. (n = 49)	p value
pSTAT3 (unstimulated at BL)	10.81 (5.27)	9.09 (2.99)	11.54 (5.63)	**0.036**
pSTAT3 (stimulated at BL)	52.46 (12.67)	57.16 (11.29)	50.86 (13.00)	0.105
pSTAT3 (unstimulated at FU)	11.45 (4.52)	9.65 (3.76)	11.85 (4.56)	0.107
pSTAT3 (stimulated at FU)	52.81 (10.99)	51.11 (10.33)	53.24 (11.35)	0.533
Diff. pSTAT3 (stimulated minus unstimulated at BL)	41.65 (11.69)	48.08 (10.19)	39.32 (11.41)	**0.012**
Delta pSTAT3 (stimulated FU minus BL)	0.867 (10.88)	6.06 (8.79)	−1.78 (11.00)	**0.018**
Delta diff. pSTAT3 (stimulated minus unstimulated at FU minus BL)	0.56 (10.26)	6.62 (7.92)	−1.65 (10.06)	**0.007**

### 3.2 STAT3 phosphorylation levels with respect to social inhibition

**Fig. 1:**
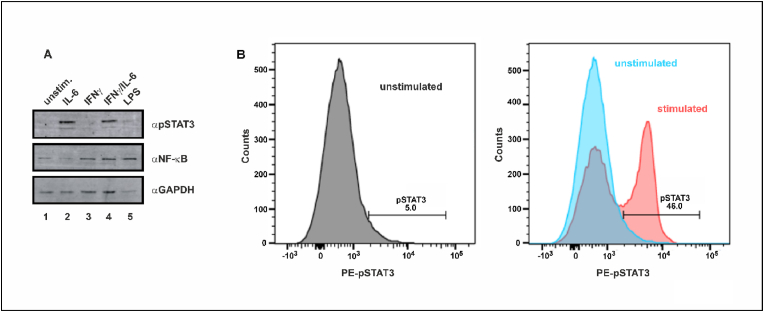
(A) Western blot analysis of tyrosine phosphorylation of STAT3 in isolated PBMCs left unstimulated (lane 1), stimulated for 45 min with 50 ng/ml IL-6 (lane 2), 50 ng/ml of interferon-γ (IFN-γ, lane 3), or the combination of both cytokines (lane 4). Stimulation with lipopolysaccharide (10 ng/ml LPS, lane 5) was used as a positive control. In addition, changes of NF-κB and glyceraldehyde 3-phosphate dehydrogenase (GAPDH) protein levels under these conditions are depicted. (B) Detection of tyrosine-phosphorylated STAT3 (Y705) by flow cytometry in PBMCs. Cells were left unstimulated (gray curve in the left graph and blue curve in the right graph) or stimulated *ex vivo* with 50 ng/ml of IL-6 (red curve in right graph) before being incubated with a phospho-tyrosine-specific antibody against STAT3. (For interpretation of the references to colour in this figure legend, the reader is referred to the Web version of this article.)

**Fig. 2:**
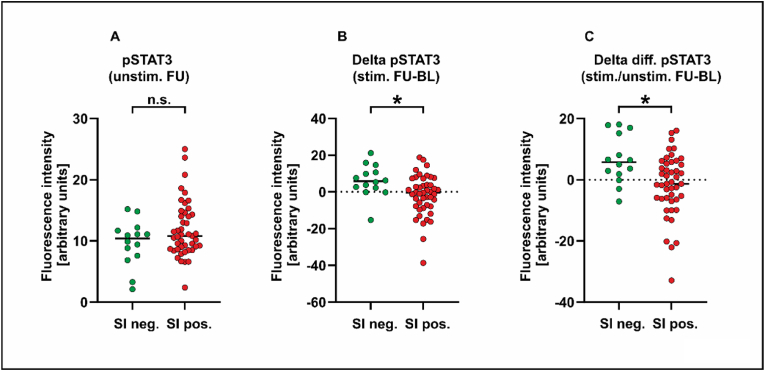
Scatter plots showing STAT3 tyrosine-phosphorylation levels measured by flow cytometry in isolated PBMCs from patients testing negative or positive for social inhibition (SI). Shown are the values for unstimulated cells (A), the difference between follow-up (FU) and baseline (BL) in IL-6-stimulated cells (B), and after subtraction from the values of the corresponding unstimulated cells (C). Abbreviations: BL; baseline, FU; follow-up, n. s.; non-significant.

Data from Western blot experiments showed that the concentration of IL-6 (50 ng/ml) used to stimulate isolated PBMCs in this study resulted in detectable tyrosine phosphorylation of STAT3 (Fig. 1A). Flow cytometry using the same tyrosine-specific anti-STAT3 antibody showed that *in vitro* IL-6 stimulation of isolated PBMCs with 50 ng/ml resulted in a significant increase in the number of pSTAT3 (Y705)-positive cells compared to unstimulated cells (Fig. 1B). Given that flow cytometry is a feasible technique to quantify *ex vivo* STAT3 activation in response to IL-6, we compared the two groups of SI-positive and SI-negative patients with respect to their STAT3 phosphorylation levels. Notably, SI-positive patients had higher levels of pSTAT3 at baseline prior to stimulation when compared to SI-negative subjects (11.5% vs. 9.1%, p = 0.036). There were no significantly different expression levels of pSTAT3 in IL-6-stimulated PBMCs at baseline and unstimulated or cytokine-stimulated cells at follow-up (Table 1, Fig. 2A). As a consequence, the increase in pSTAT3 levels from unstimulated to stimulated cells differed significantly between the two SI groups, with PBMCs from SI-negative subjects being more sensitive to IL-6 stimulation (48.1% vs. 39.3%, p = 0.012). After five to six weeks of inpatient psychotherapy, the mean total pSTAT3 level in IL-6-stimulated cells increased in SI-negative patients, whereas it decreased for SI-positive patients (6.1% vs. −1.8%), again resulting in a significant difference between the two groups (p = 0.018) (Fig. 2B). The same pattern was observed for the cellular capacity for IL-6 stimulation (6.6% vs. −1.7%), which also reached statistical significance (p = 0.007) (Fig. 2C). No significant associations were observed between pSTAT3 and NA, as the second component of the type D personality (data not shown). In summary, SI-positive and SI-negative patients differed with respect to their STAT3 phosphorylation levels in isolated PBMCs both at study inclusion and hospital discharge.

### 3.3 Correlations between social inhibition and STAT3 phosphorylation

**Fig. 3:**
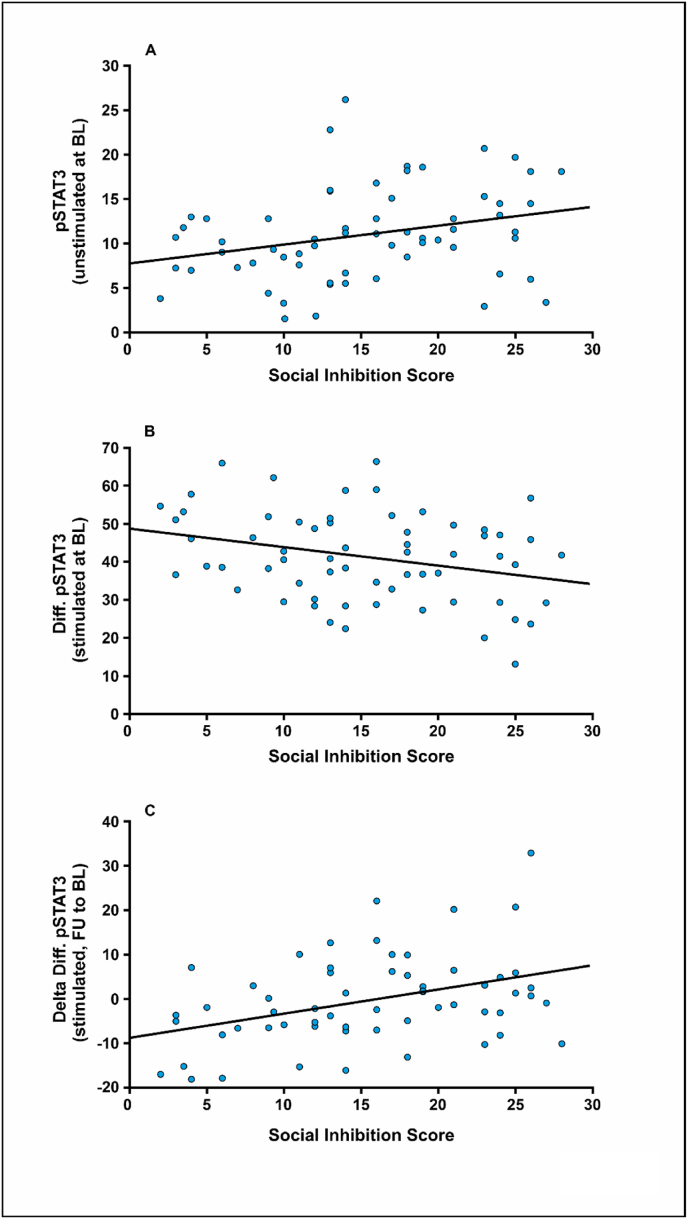
Correlations between pSTAT3 levels in PBMCs isolated from depressed patients under unstimulated and stimulated conditions (50 ng/ml IL-6) and the social inhibition (SI) score, as determined by the DS-14 instrument. The SI score was correlated to (A) pSTAT3 levels determined by flow cytometry in PBMCs obtained at hospital admission prior to cytokine exposure, (B) the difference between pSTAT3 levels in stimulated to unstimulated cells at baseline (BL), or (C) the difference of pSTAT3 stimulability in the course of psychotherapy (from BL to follow-up at hospital discharge).

**Fig. 4:**
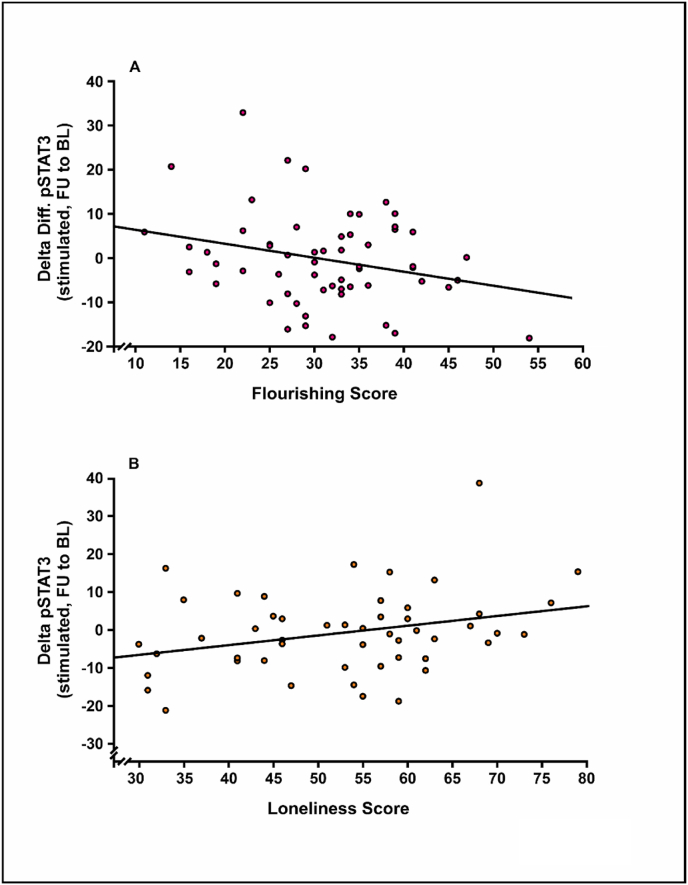
Associations between STAT3 phosphorylation in isolated PBMCs stimulated *ex vivo* with IL-6 and the psychometrically assessed scores from the Flourishing and the UCLA Loneliness scales. (A) Patients with a higher Flourishing score at baseline showed a reduced STAT3 inducibility of isolated PBMCs. (B) The Loneliness score correlated positively with changes in the STAT3 phosphorylation level upon cytokine stimulation.

Pearson's correlation analysis was used to test for significant relationships between the SI score and the pSTAT3 levels in the patients' PBMCs. Our data showed that, in unstimulated PBMCs at baseline, STAT3 phosphorylation was significantly and positively correlated to the SI score (r = 0.287, p = 0.023) (Fig. 3A). The PBMCs´ responsiveness to IL-6 stimulation was negatively correlated with the HADS SI score (r = −0.295, p = 0.019) (Fig. 3B). In the course of hospital treatment, the stimulability of STAT3 was positively associated with HADS SI (r = 0.388, p = 0.002) (Fig. 3C) and negatively with the sum score from the Flourishing Scale (r = −0.267, p = 0.043) (Fig. 4A). However, similar associations could not be observed for the other component of type D personality, namely NA (data not shown). A significant, positive correlation was found between IL-6-induced STAT3 phosphorylation and the UCLA loneliness score during hospital treatment (r = 0.300, p = 0.033) (Fig. 4B). In conclusion, we observed that the flow cytometrically measured STAT3 phosphorylation levels in IL-6-stimulated PBMCs correlated significantly with the SI score, as was the case for the UCLA loneliness score.

### 3.4 Associations of SI and pSTAT3 in a multivariate analysis

A series of logistic regression models were computed to determine whether SI was associated with the level of STAT3 phosphorylation in PBMCs, when adjusted to the potentially confounding variables sex, age, diabetes mellitus and hypertension. The two latter parameters differed significantly between the SI-positive and -negative group, and were therefore included in the models. Data showed that the level of IL-6-stimulated pSTAT3 at follow-up was associated with an elevated psychometrically assessed SI score (Exp(β) = 1.33, 95%-CI = 1.04; 1.71, p = 0.024; Model 1 in Table 2). The assignment to the SI groups was associated with the stimulability of the PBMCs at follow-up (Exp(β) = 1.098, 95%-CI = 1.00; 1.20, p = 0.045; Model 2). During hospital treatment, pSTAT3 expression in IL-6-stimulated PBMCs differed significantly between the two SI groups (Exp(β) = 0.91, 95%-CI = 0.83; 0.99, p = 0.037; Model 3), as was also observed for the difference between stimulated and unstimulated PBMCs (β = 0.89, 95%-CI = 0.80; 0.99, p = 0.029; Model 4). When antidepressant pharmacotherapy was included as an additional confounder, this result did not change (p = 0.033).

**Table 2:** Results from four binary logistic regression models with social inhibition as the dependent variable adjusted for the following confounders: sex, age, diabetes mellitus, hypertension and phosphorylation of STAT3. Significant changes are highlighted in bold.

	Model 1 (R^2^ = 0.293; p = 0.001)		
Variable	Exp(β)	95% CI	p value
Sex	0.302	0.051; 1.794	0.188
Age	1.089	1.004; 1.181	**0.039**
Diabetes mellitus	0.008	0.000; 0.662	**0.032**
Hypertension	0.085	0.007; 1.041	0.054
pSTAT3 (stimulated at FU)	1.333	1.039; 1.709	**0.024**
	Model 2 (R^2^ = 0.269; p = 0.002)		
Variable	Exp(β)	95% CI	p value
Sex	0.173	0.023; 1.276	0.085
Age	1.115	1.016; 1.224	**0.022**
Diabetes mellitus	0.033	0.001; 0.746	**0.032**
Hypertension	0.051	0.004; 0.727	**0.028**
pSTAT3 (stimulated minus unstimulated at FU)	1.098	1.002; 1.202	**0.045**
	Model 3 (R^2^ = 0.280; p = 0.002)		
Variable	Exp(β)	95% CI	p value
Sex	0.339	0.064; 1.800	0.204
Age	1.057	0.991; 1.128	0.092
Diabetes mellitus	0.075	0.004; 1.562	0.095
Hypertension	0.108	0.012; 1.015	0.052
Delta pSTAT3 (stimulated at FU minus BL)	0.910	0.832; 0.994	**0.037**
	Model 4 (R^2^ = 0.291; p = 0.001)		
Variable	Exp(β)	95% CI	p value
Sex	0.371	0.074; 1.867	0.229
Age	1.062	0.992; 1.136	0.084
Diabetes mellitus	0.167	0.010; 2.926	0.221
Hypertension	0.088	0.009; 0.908	**0.041**
Delta diff. pSTAT3 (stimulated minus unstimulated at FU minus BL)	0.891	0.804; 0.988	**0.029**

Abbreviations: BL; baseline, FU; follow-up.

## 4 Discussion

The CitDip study investigates the activation of the IL-6-inducible transcription factor STAT3 in PBMCs from depressed inpatients in the course of a psychotherapeutic intervention and demonstrates that the level of STAT3 tyrosine phosphorylation was significantly associated with a subject's tendency to display SI at baseline. PBMCs from socially inhibited study participants had a reduced capacity to be externally stimulated by the STAT3-activating cytokine IL-6, possibly due to their high initial STAT3 phosphorylation. Moreover, our data show that changes in the STAT3 stimulability measured in the course of psychotherapeutic treatment were significantly and positively correlated with the psychometrically assessed SI score. In addition, our data demonstrate that loneliness, a negative emotional state related to perceptions of SI and/or social exclusion, was correlated with changes in STAT3 phosphorylation during hospital treatment. Regression models adjusted for clinically relevant confounding factors confirmed the result of the univariate analysis that IL-6-induced STAT3 activation was associated with a socially inhibited personality profile in depressed patients.

A meta-analysis has linked loneliness and social isolation to IL-6 (Smith et al., 2020), which is a well-established inflammatory marker and direct activator of the JAK-STAT3 signal pathway (MacAulay et al., 2024). However, research on the relationship between STAT3 activation and SI or loneliness has not been reported in the literature so far. Some studies have postulated a link between STAT3 signaling and depressive symptoms. Associations were found between polymorphisms in either the *IL-6* or serotonin transporter (*SERT)* gene and the development of depressive symptoms (Udina et al., 2013; Meyer et al., 2020). Cytokine-induced STAT activation results in increased expression of indoleamine 2,3-dioxygenase (IDO), which is the key enzyme in the degradation of tryptophan to kynurenine. The STAT3-mediated elevation in IDO expression results in a reduced concentration of the neurotransmitter serotonin, which may contribute to the pathogenesis of MDD and/or social withdrawal (Taylor and Feng, 1991; Müller and Schwarz, 2007).

Animal studies have indicated that the IL-6/STAT3 signaling pathway is involved in depression-like behaviours (Guan et al., 2021) and that STAT3 controls IL-6-dependent *SERT* expression (Kong et al., 2015). Furthermore, mice carrying a tissue-specific microglial or midbrain disruption of the *Stat3* gene displayed reduced emotional behaviours under stress as well as antidepressive-like behaviour (Kwon et al., 2017; Reisinger et al., 2021). Additionally, there is indirect evidence of a possible antidepressant effect of JAK inhibitors in animals and humans (Shariq et al., 2018). At last, IL-6 knock-out mice were reported to show reduced despair and diminished learned helplessness in forced swim and tail suspension tests, as well as enhanced hedonic behaviour in a sucrose preference test (Chourbaji et al., 2006). Taken together, these data demonstrate that the IL-6/STAT3 signaling pathway plays an important role in depression or depression-like behavious, and targeting this pathway may provide a novel therapeutic approach for the treatment of this disorder.

Although limited evidence suggest that STAT proteins are linked to depression in the literature, data on a putative association of this transcription factor with the type D personality or social withdrawal are not available. In particular, prospective studies on possible immunomodulatory effects of psychotherapeutic intervention have rarely been performed in clinical settings so far. Data from the SPIRR-CAD study demonstrated that depressive patients with coronary heart disease benefit more from psychotherapeutic treatment if they were assigned to type D personality (Herrmann-Lingen et al., 2016). To better understand the biological mechanisms underlying this finding, we performed the presented study in which we investigated the *in vitro* stimulatory capacity of PBMCs, defined as the differences in pSTAT3 levels of IL-6-stimulated versus unstimulated cells during five to six weeks of hospitalization.

STAT proteins are usually activated in the context of an inflammatory reaction, and particularly the founding member of this family, the interferon-inducible STAT1, constitutes the first line of defence against a broad spectrum of pathogenic microorganisms by mounting an interferon-induced antimicrobial response. Pharmacological treatment of viral hepatitis with interferons significantly reduces viral load, while leading to a dose-dependent induction of depressive symptoms and sickness behaviour as major side effects (Hauser et al., 2002; Udina et al., 2012). Moreover, suicidal depressives were found to have increased expression of depression-related interferon-induced genes, presumably regulated by STAT1, compared to healthy individuals (Hoyo-Becerra et al., 2013). Since STAT1 and STAT3 generally exert antagonistic functions via the induction of genes with opposing effects, an imbalance in the activation of these transcription factors could influence the patient's behaviour (Ho and Ivashkiv, 2006; Stephanou and Latchman, 2005). A recently published proteome-wide analysis by Gong et al. has shown that loneliness and social isolation, both linked to social deficit behaviour, have similar protein signatures, including the regulation of programmed cell death, caspase activation, malignant transformation, and host susceptibility to infection (Gong et al., 2025). All these pathways are known to be upregulated by STAT proteins (Doudin et al., 2023), which in cytokine-exposed cells bind as phosphorylated dimers sequence-specifically to promoter regions termed GAS [gamma-activated sites] elements and subsequently modulate transcription of genes by recruiting polymerase II.

The fact that STAT3-regulated gene expression signatures resemble those that are upregulated in socially isolated individuals suggests that these transcriptional activators may be the missing molecular link between cytokine activation and social deficit behaviours such as loneliness, social isolation, or social withdrawal. The stimulatory capacity of the cells was significantly lower in the group of SI-positive patients compared to their SI-negative counterparts. Their lower stimulability of STAT3 suggests that the activation of this proliferative and anti-apoptotic pathway may be less sensitive in PBMCs isolated from SI-positive patients. The reduced activation of STAT3 in individuals with marked SI observed in our study would be consistent with the hypothesis that the inducibility of the STAT3 signaling pathway is blunted in these patients during psychotherapeutic intervention. In individuals with social inhibition, the STAT3 signaling pathway may be partially inhibited, resulting in a decreased proliferative response to cytokine stimulation. Therefore, in depressed subjects with social deficit behaviours, the attenuated response of STAT3 to changes in extracellular IL-6 concentration may represent a potential future therapeutic strategy.

It is noteworthy that our study has several limitations. These include the relatively small sample size of the patient cohort, the lack of a group of healthy or untreated control subjects, and the non-randomized design of the trial. Due to the observational nature of this study, no causal relationship can be deduced from our findings. In addition, there was a heterogeneity in terms of medication and prior treatment among the included subjects, which may have influenced the results. In the studied population, 64% of subjects were taking antidepressants at study inclusion and 63% reported a history of prior psychotherapy. Patients pretreated with antidepressants might already have a blunted inflammatory cytokine activity, and other pharmacological therapies might also interfere in an immunomodulatory manner affecting STAT3 activation. Patients previously treated with antidepressants could be subject to chronic recurrent depression or treatment resistance, which is associated with an altered inflammatory profile (Strawbridge et al., 2015; Arteaga-Henríquez et al., 2019; Chamberlain et al., 2019). A twofold assessment of the depression severity can be positively emphasized, which was achieved through clinical interviews by mental health professionals at hospital admission and the self-reported HADS questionnaire as an established screening instrument.

In summary, our results describe for the first time a relationship between SI and the IL-6-induced stimulatory capacity of the STAT3-expressing PBMCs in the context of depression. The stimulatory capacity of the cells, defined as the differences in pSTAT3 levels from IL-6-stimulated and unstimulated cells during the five to six weeks of hospitalization, was significantly higher in the SI-negative group than in the SI-positive patients. From these data, we suggest that the SI personality trait cannot be regarded exclusively as a psychological construct, but is likely also linked to systemic changes in intracellular cytokine signaling in PBMCs.

## CRediT authorship contribution statement

**Katharina von Knebel:** Writing – review & editing, Methodology, Investigation, Formal analysis, Data curation. **Julia Staab:** Writing – review & editing, Methodology, Investigation, Formal analysis. **Anke Gregus:** Writing – review & editing, Methodology. **Linus Remling:** Writing – review & editing, Writing – original draft, Methodology. **Oliver Wirths:** Writing – review & editing, Methodology. **Carsten Spitzer:** Writing – review & editing, Methodology, Conceptualization. **Christoph Herrmann-Lingen:** Writing – review & editing, Methodology, Conceptualization. **Holger M. Reichardt:** Writing – review & editing, Writing – original draft, Methodology, Formal analysis, Conceptualization. **Thomas Meyer:** Writing – review & editing, Writing – original draft, Methodology, Formal analysis, Conceptualization.

## Declaration of competing interest

The author Christoph Herrmann-Lingen declares conflicts of interest: He is receiving royalties from Hogrefe Publishers for the German version of the Hospital Anxiety and Depression Scale. During the last 3 years, he has received a lecture honorarium from Novartis. The following authors declare they have no conflicts of interest: Katharina von Knebel, Julia Staab, Anke Gregus, Linus Remling, Oliver Wirths, Carsten Spitzer, Holger M. Reichardt, and Thomas Meyer. The authors report no source of funding for this work.

## Data Availability

Data will be made available on request.
